# HMGB1 promotes the activation of NLRP3 and caspase-8 inflammasomes via NF-κB pathway in acute glaucoma

**DOI:** 10.1186/s12974-015-0360-2

**Published:** 2015-07-30

**Authors:** Wei Chi, Hongrui Chen, Fei Li, Yingting Zhu, Wei Yin, Yehong Zhuo

**Affiliations:** State Key Laboratory of Ophthalmology, Zhongshan Ophthalmic Center, Sun Yat-sen University, 3#925 Xianlienan Road, Guangzhou, 510060 China; Zhongshan medical college, Sun Yat-sen University, Guangzhou, 510030 China

**Keywords:** Acute glaucoma, HMGB1, NLRP3 inflammasome, Caspase-8 inflammasome

## Abstract

**Background:**

Acute glaucoma is a significantly sight-threatening cause of irreversible blindness in the world characterized by a sudden and substantial intraocular pressure (IOP) increase and subsequent retinal ganglion cell (RGC) death. This study aims to explore the role of high-mobility group box 1 (HMGB1) in an acute glaucoma mouse model.

**Methods:**

An acute glaucoma model was induced by a rapid and substantial increase IOP to 70 mmHg for 60 min via anterior chamber punctured and affused with Balance Salt Solution in C57BL/6 mice. Retinal tissue ischemic damage and loss of RGCs were assessed at 6, 24, 48, 72 h after high IOP treatment, and at 48 h, group with or without recombinant high-mobility group box 1 (rHMGB1), the HMGB1 inhibitor, glycyrrhizic acid (GA), and by HE and immunofluorescent staining. The nuclear factor κB (NF-κB) inhibitor, JSH-23, and caspase-8 inhibitor, Z-IETD-fmk, were injected into vitreous. Reverse transcription and semi-quantitative reverse transcription polymerase chain reaction (RT-PCR), western blotting, and immunoprecipitation were performed to evaluate the expression level of nucleotide-binding domain, leucine-rich repeat containing protein 3 (NLRP3), phosphor-NF-κB p65, caspase-8, caspase-1, apoptosis-associated speck-like protein containing a CARD (ASC), and interleukin-1β (IL-1β).

**Results:**

HMGB1 was increased in ischemic retinal tissue during acute glaucoma as early as 6 h after rapid IOP elevation. Exogenous HMGB1 exacerbated retinal ischemic damage, RGC loss, and inhibition of endogenous HMGB1 significantly reduced the severity of disease. HMGB1 significantly induced the elevation of canonical NLRP3, ASC, caspase-1, and non-canonical capase-8-ASC inflammasome and promoted the processing of IL-1β. Furthermore, the effect of HMGB1 on NLRP3 inflammasome activation and IL-1β production was dependent on NF-κB pathway. Thus, HMGB1/caspase-8 pathway promoted the processing of IL-1β via NF-κB pathway.

**Conclusion:**

The findings of this study identified a novel signaling pathway in which HMGB1, in response to acutely elevated intraocular pressure, activated the canonical NLRP3 and non-canonical caspase-8 inflammasomes and production of IL-1β during acute glaucoma development. These results provide new insights to the understanding of the innate response that contributes to pathogenesis of acute glaucoma.

## Background

Glaucoma is the leading cause of permanent vision loss and irreversible blindness in the world [1]. Acute glaucoma is caused by a blockage around the trabecular meshwork allowing buildup of aqueous humor which results in a rapid increase in intraocular pressure (IOP). This causes retinal ischemic reperfusion (IR) injury and retinal ganglion cell (RGC) death [2]. The precise mechanisms by which elevated IOP leads to RGC death are unclear absolutely. High-mobility group box 1 (HMGB1) protein is an abundant protein that has been shown to be involved in the pathogenesis of several inflammatory diseases, including elevated IOP-induced of the inherited glaucoma rat model [3–5]. Once released by necrotic cells in the extracellular milieu, HMGB1 functions as an alarming or a damage-associated molecular pattern (DAMP) [6, 7]. Several reports have demonstrated that HMGB1 mediates ischemia-associated inflammatory responses, inducing damage in several IR diseases [8–11]. Extracellular HMGB1 induces IR inflammatory responses by directly acting on pattern recognition receptors, including Toll-like receptors (TLR) 2 and 4 and receptors for advanced glycation end products (RAGEs) [12–14].

Inflammasomes are intracellular multi-protein cytoplasmic complexes that play a central role in several IR diseases [15–18]. Canonical inflammasomes typically are multi-protein assemblies formed by the adaptor protein apoptosis-associated speck-like protein containing a CARD (ASC), effector caspase-1, and NOD-like receptor (NLR) family or absent in melanoma 2 (AIM2) [19, 20]. In IR diseases, DAMPs trigger the activation of NLRs which oligomerize to form a platform for the inflammasome, leading to processing of pro IL-1β into its mature forms via caspase-1 activity [21–23]. Previous reports have shown nucleotide-binding domain, leucine-rich repeat containing protein 3 (NLRP3) inflammasome to be involved in IR injury by promoting the release of HMGB1 [24, 25]. However, the contribution of HMGB1 to NLRP3 inflammasome activation has not been explored in IR injury. In order to investigate the effect of HMGB1 on the activation of NLRP3 inflammasome in acute glaucoma, we injected exogenous recombinant (r) HMGB1 and anti-HMGB1 antibody into vitreous in our acute glaucoma model. We hypothesized that HMGB1 would promote the activation of NLRP3 inflammasome to mediate retinal ischemic damage and RGC death.

Caspase-8, an initiator caspase with a critical role in triggering cell apoptosis, is synthesized as a pro-enzyme and contains a large N-terminal prodomain and a C-terminal catalytic domain composed of a large and small subunit separated by a small linker [26]. Our previous study showed that elevated IOP could induce the activation of TLR4-caspase-8 pathway which led to retinal ischemic damage and RGC death [27]. Since HMGB1 is the endogenous ligand of TLR4, we therefore hypothesized that HMGB1 may be involved in acute glaucoma via regulating caspase-8 activation and the process of IL-1β. Greater understanding of HMGB1 in acute glaucoma may lead to new therapeutic targets that may halt the retinal ischemic damage and RGC death.

## Methods

### Ethics statement

C57BL/6 female mice of 6–8 weeks of age were purchased from the Animal Research Center at Zhongshan University. The care and use of animals adhered to the Association for Research in Vision and Ophthalmology (ARVO) Statement for the use of Animals in Ophthalmic and Vision Research and were approved and monitored by the Institute of Committee of Animal Care of the Zhongshan Ophthalmic Center (Permit Number: 2011–038).

### Establishment of retinal IR model

Mice were anesthetized with 10 ml/kg of 4.3 % chloral hydrate by intraperitoneal injection. Pupils were dilated with 1 % tropicamide and corneas were anesthetized topically with 0.5 % tetracaine hydrochloride eye drops. Cannulation of the anterior chamber of the right eye with a 30-gauge needle was performed to increase and maintain IOP at 70 mmHg by addition of Balance Salt Solution (Tono-Pen XL; Medtronic Solan, Jacksonville, FL, USA) for 60 min. The left eye served as a control. After 60 min, the needle was withdrawn and tobramycin was applied to avoid bacterial infection. Mice were sacrificed at 6, 24, 48, or 72 h after the procedure.

### Intravitreal injection

After induction of IR injury, mice received an intravitreal injection of recombinant human HMGB1 (1 μg/2 μl; 1690-HM-025, R&D system, Inc., USA), glycyrrhizic acid (120 μM/2 μl; 50531, Sigma Aldrich, USA), nuclear factor κB (NF-κB) inhibitor JSH-23 (20 μM/2 μl; J4455, Sigma Aldrich, USA) , caspase-8 inhibitor Z-IETD-fmk (20 μM/2 μl; Calbiochem, San Diego, CA, USA), and PBS (2 μl) vehicle as sham. Mice were sacrificed and eyes were enucleated 48 h after intravitreal injection.

### Histological examination

At the experimental time points, mice were sacrificed and eyes were enucleated and fixed with 4 % paraformaldehyde overnight prior to paraffin embedding. Four 4-mm-thick sections through the optic nerve of each eye were cut and stained with hematoxylin and eosin (HE). Total retinal thickness (from inner to outer limiting membrane, ILM-OLM) was measured in four adjacent areas within 1 mm distance to the optic nerve center using Axiovision software (Carl Zeiss MicroImaging Inc., Thornwood, NY, USA). The samples for confocal immunofluorescent staining were embedded in an optimal cutting temperature compound (OCT) (Tissue-Tek, Sakura Finetek USA, Torrance, CA, USA) and stored at −80 °C for frozen sectioning. The section was made in 6 μm, blocked and permeated with 5 % BSA-0.5 % Triton for 1 h at room temperature (RT), and incubated with primary antibody to detect cleaved-caspase-8 (1:500; #8592, Cell Signaling Technology, Beverly, MA, USA) at 4 °C overnight. Alexa Fluor 488 donkey anti-rabbit IgG (A-21206, Invitrogen, Carlsbad, CA, USA; 1:400) was used to visualize the primary antibody. Nuclei were stained with 4′, 6-diamidino-2-phenylindole dihydrochloride (D1306, DAPI, Invitrogen, Carlsbad, CA, USA). 400× images were collected and analyzed with a confocal microscope (Carl Zeiss, Inc, Germany). Statistical analysis was used to compare the differences among sham, high-mobility group box 1 (rHMGB1), glycyrrhizic acid (GA), IR + PBS, IR+ rHMGB1 and IR + GA groups.

### Retinal flat and RGCs quantification

Retinal flat mounts from each group were fixed with 4 % paraformaldehyde for 15 min at RT, rinsed with PBS three times and blocked and permeated with 20 % BSA-0.5 % Triton-X 100 at RT for 2 h. To detect RGC markers, flat mounts were incubated overnight at 4 °C with 1:200 anti-β3-tubulin primary antibody (#5568, poly rabbit anti β3-tubulin, Cell Signaling Technology, Beverly, MA, USA). Secondary antibody was used as frozen section for confocal microscope. Images were collected and analyzed with a fluorescent microscope (Carl Zeiss, Inc, Germany). Four 400× images were obtained from each of the four quadrants of whole retinal flat mounts. The intensity of fluorescence was analyzed by using Image Pro Plus (Version 6.0; Media Cybernetics).

### Semi-quantitative reverse transcription-polymerase chain reaction

Total RNA was extracted from retina samples using Trizol Reagent (Invitrogen, Carlsbad, CA, USA). cDNA was synthesized with PrimeScript RT Master Mix (DRR036A, TaKaRa, Dalian, China). PCR was carried out using a Premix EX Taq Kit (D335A, TaKaRa, Dalian, China) for 25 cycles of GAPDH and 28–30 cycles of the other target genes. PCR products were run on a 1.5 % agarose gel, and gene expression was evaluated by relative pixel densitometry using Image J software (National Institutes of Health, USA) after normalization to GAPDH. The primer sequences are as follows: caspase-8 (forward, 5-ctccgaaaaatgaaggacaga-3; reverse, 5-cgtgggataggatacagcaga-3), nlrp3 (forward, 5-ggtcctctttaccatgtgcttc-3; reverse, 5-aagtcatgtggctgaagctgta-3), asc (forward, 5-cttgtcaggggatgaactcaa-3; reverse: 5-ctggtccacaaagtgtcctgt-3), il-1beta (forward, 5-tgaaatgccaccttttgacag-3; reverse, 5-ccacagccacaatgagtgatac-3), gapdh (forward, 5-aggtcatcccagagctgaacg-3; reverse: 5-caccctgttgctgtagccgtat-3).

### Western blot analysis

Total and cytoplasmic protein was isolated from retina samples. Proteins were run on 10 or 12 % polyacrylamide gels and transferred to polyvinylidene difluoride (PVDF) membranes. PVDF membranes were blocked with 5 % BSA at RT for 60–90 min and incubated overnight at 4 °C with antigen-specific primary antibodies. Blots were then incubated with species-specific HRP-conjugated secondary antibodies for 60 min at RT. Proteins were visualized by incubation with a chemiluminescence substrate kit (ECL Plus; Perkin Elmer Inc., Covina, CA, USA). The expression of target proteins was quantified by Quantity One software (The Discovery Series) after normalizing to β-actin or GAPDH.

The primary antibodies and dilutions were used as follows: NLRP3 (1:500; NBP1-77080, Novus, Littleton, CO, USA, 100 kD), ASC (1:500; 04–147, Millipore, Temecula, CA, USA, 22 kD), phosphor-NF-κB p65 (1:1000; #3033, Cell Signaling Technology, Beverly, MA, USA, 65 kD), cleaved caspase-8 (1:500; #8592, Cell Signaling Technology, Beverly, MA, USA, 18 kD), caspase-1 (1:200; AB1871, Chemicon, International, Inc., USA, pro-caspase-1 45 kD, cleaved-caspase-1 20 kD), IL-1β (1:500; #8689, Cell Signaling Technology, Beverly, MA, USA, pro-IL-1β 31 kD, IL-1β 17 kD), β-actin (1:1000; MAB1445, MultiSciences Biotech, Hangzhou, China, 45 kD), and GAPDH (#2118, Cell Signaling, Boston, MA, USA, 36 kD).

### Immunoprecipitation

Total protein was extracted from each group as stated above and stored at −80 °C. Incubated with 1 μl anti-caspase-8 or ASC antibody overnight at 4 °C was 50 μl of protein. This reaction mixture was then incubated with protein A magnetic beads (2366538, Millipore, Temecula, CA, USA) for 30 min at 4 °C. Precipitates were washed three times with washing buffer and then eluted from protein A magnetic beads by boiling with 1 × SDS for 10 min at 90–100 °C. Western blot analysis was used to evaluate the expression of caspase-8 and ASC. Immunoprecipitation antibody, anti-caspase-8 (#8592, Cell Signaling Technology, Beverly, MA), anti-ASC (sc-22513,Santa Cruz, CA, USA); Western blot analysis anti-caspase-8 (sc-6134, Santa Cruz, CA, USA), anti-ASC (ab175449, Abcam), homophytic IgG as the negative control.

### Statistical analysis

The data were presented as mean ± SD or percentage. One-way ANOVA, followed by the Dunnett’s multiple comparison tests and two-way ANOVA were performed using GraphPad Prism software (version 5.0, GraphPad Software, Inc., San Diego, CA, USA). All statistical assessments were two-sided, and *P* values less than 0.05 were considered statistically significant.

## Results

### The addition of HMGB1 increased severity of disease, whereas the inhibition of HMGB1 decreased severity of acute glaucoma

**Fig. 1:**
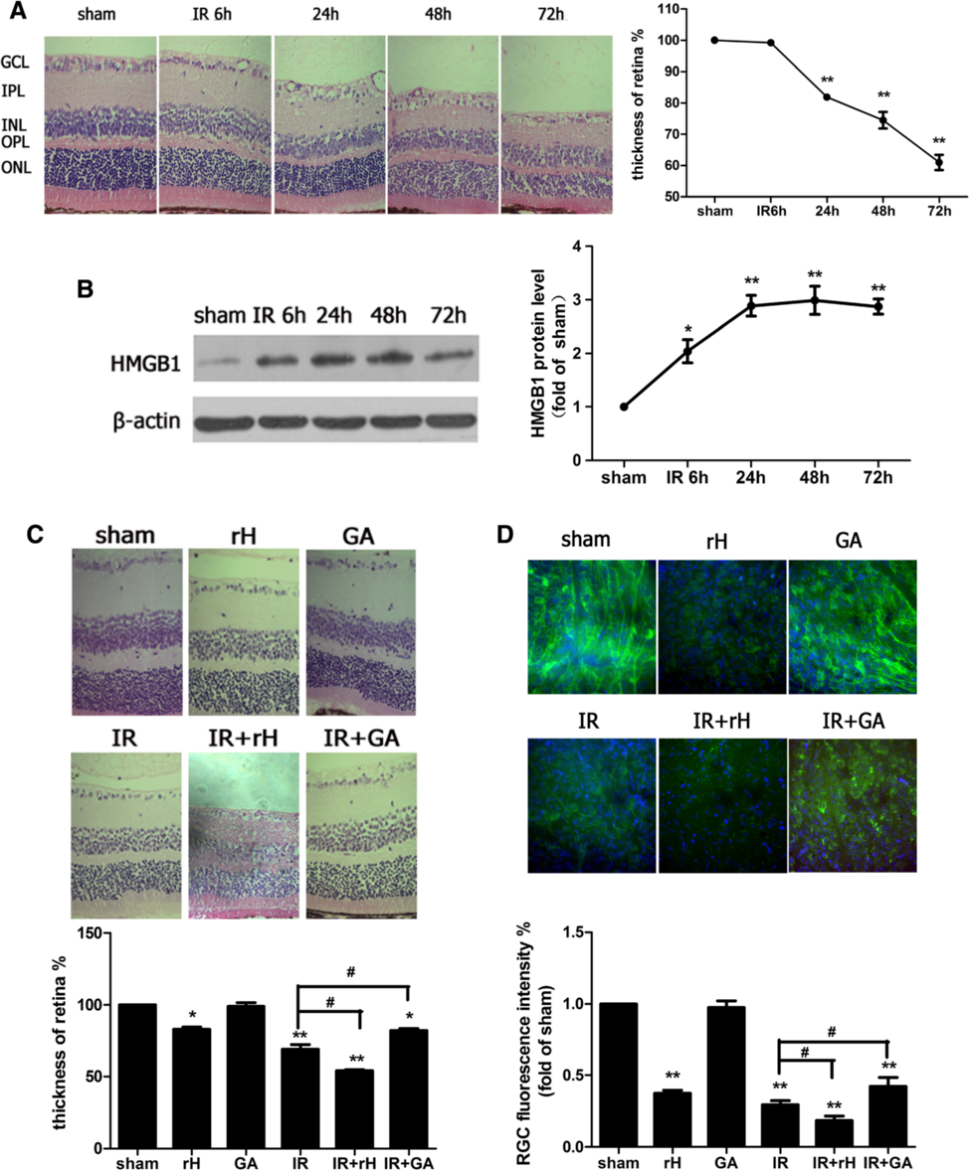
HMGB1 was actively involved in the pathogenesis of retinal IR injury. **a** Hematoxylin and eosin staining of retina showed the degeneration of RGCs and mean thickness decrease of ischemic retinal tissue at different time points after reperfusion. *GCL* ganglion cell layer, *IPL* inner plexiform layer, *INL* inner nuclear layer, *ONL* outer nuclear layer, *OPL*, outer plexiform layer. **b** Western blot analysis of the cytoplasmic protein levels of HMGB1 at different time points after reperfusion. Each protein expression level is shown relative to that of controls. **c** The function of HMGB1 on the retinal tissue damage in retinal IR injury. The sham procedure was performed without elevating the container in contralateral eyes as control groups and retinal ischemia reperfusion (IR) injury was observed at 48 h after reperfusion. ^#^ *P* < 0.05 (IR vs IR + rH, IR vs IR + GA). **d** RGCs marker β3-tubulin detected RGCs damage by immunofluorescent staining in experimental groups. Data are shown as mean ± SD or percentage. *P < 0.05, **P < 0.01 vs sham group

DAMPs trigger the release of HMGB1 in response to IR damage [28, 29]. In this study, we sought to determine the role of HMGB1 in the development of retinal IR injury caused by elevated IOP radically. In retinal IR injury models, retinal damage occurs rapidly with retinal edema, vacuolar degeneration, and condensation of nuclear chromatin. HE staining showed that the retinal thickness was decreased as early as 24 h after reperfusion (Fig. 1a). Additionally, retinal ischemia damage rapidly initiated the release of HMGB1 at 6 h after reperfusion and peaked at 48 h in the protein level (Fig. 1b). Stimulation of HMGB1 increased the severity of retinal thickness reduction, and the number of RGC death was exacerbated, by contrast, the inhibition of HMGB1 decreased the reduction of retinal thickness and the number of RGCs death in the retinal IR injury models (Fig. 1c, d). These results indicated the pivotal role of HMGB1 in mediating retinal ischemic damage and RGC death.

### HMGB1 promoted caspase-8 inflammasome activation in acute glaucoma

**Fig. 2:**
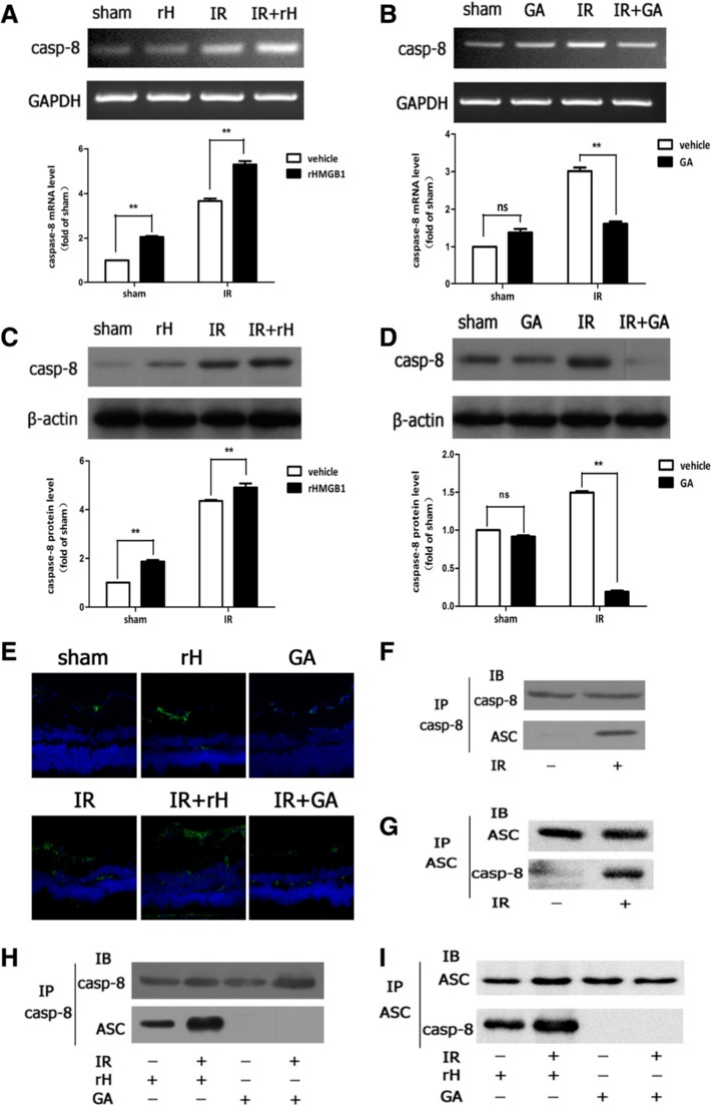
HMGB1 promoted non-canonical caspase-8 inflammasome assembled in retinal IR injury. **a**, **b** RT-PCR analyzed caspase-8 mRNA expression. **c**, **d** Western blot detected expression of cleaved caspase-8 protein. **e** rHMGB1 could stimulate the activation of caspase-8 in RGCs, while inhibition of HMGB1 significantly suppressed the activation by confocal microscope. **f**, **g** ASC immunoprecipitated with caspase-8 and assembled caspase-8-ASC complex in IR model. **h**, **i** HMGB1 promoted caspase-8-ASC protein complex formation. Data are shown as mean ± SD. *P < 0.05, **P < 0.01 vs sham group

Caspase-8 has been implicated as an initiator caspase in death receptor-induced signaling of apoptosis [26]. However, we recently reported a non-apoptotic function of caspase-8 in mediating inflammatory damage in retinal IR injury [27]. In our study, we clarified that exogenous rHMGB1 promoted the protein up-regulation of cleaved caspase-8 in retinal IR injury. Inhibition of HMGB1 significantly suppressed the expression of cleaved caspase-8 in ischemia retinal tissue (Fig. 2a–e). Other recent reports have demonstrated novel caspase-8 inflammasome activation in response to bacterial or fungal infection [30, 31]. Therefore, we hypothesized that a non-canonical caspase-8 inflammasome was involved in the development of retinal IR injury. Caspase-8 was immunoprecipitated with ASC, the adaptor protein in most inflammasomes, suggesting a role for caspase-8 in ischemic retinal tissue (Fig. 2f, g). And rHMGB1 was found to be relevant to the formation of non-canonical caspase-8 inflammasome (Fig. 2h, i). This finding suggested that a non-canonical caspase-8 inflammasome was involved in the mechanisms of retinal IR damage.

### HMGB1 regulated the activation of canonical NLRP3 inflammasome in elevated IOP-induced retinal IR injury

Previous studies have reported that NLRP3 inflammasome activation promotes the release of HMGB1 and inflammatory cytokines, such as IL-1β and IL-18, in several IR diseases [24, 25]. However, the effect of HMGB1 on NLRP3 inflammasome activation in retinal IR injury has not been examined. Therefore, we tested the influence of HMGB1 on the activation of the most characterized inflammasome, NLRP3 inflammasome, in retinal IR injury.

**Fig. 3:**
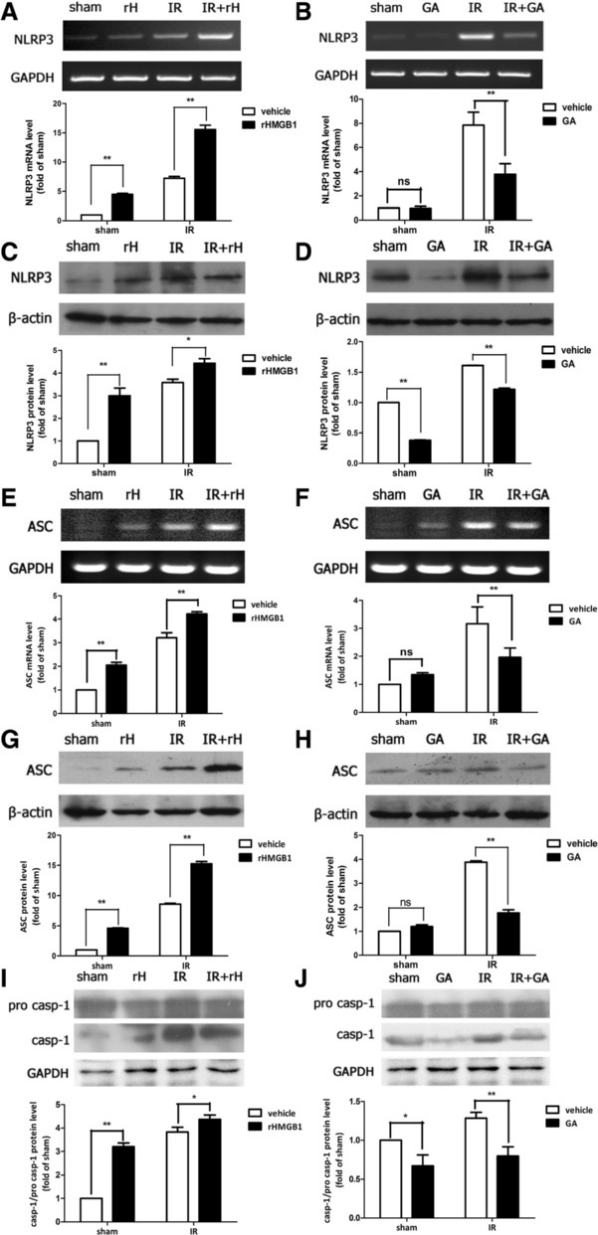
HMGB1 promoted the activation of canonical NLRP3 inflammasome in retinal IR injury. The intravitreal injection of exogenous rHMGB1 significantly promoted the mRNA and protein production of NLRP3 (**a**, **c**) and ASC (**e**, **g**) and activation of caspase-1 (**i**). Thus, the intravitreal injection of HMGB1 inhibitor, GA, significantly suppressed the production of NLRP3 (**b**, **d**) and ASC (**f**, **h**) and activation of caspase-1 (**j**). Data are shown as mean ± SD. *P < 0.05, **P < 0.01 vs sham group

Our findings demonstrated that exogenous rHMGB1 promoted the protein up-regulation of NLRP3, ASC level, and increased caspase-1 processing after reperfusion in retinal IR injury (Fig. 3a, c, e, g, i). Inhibition of HMGB1 significantly suppressed the expression of NLRP3, ASC, and the maturation of caspase-1 in ischemia retinal tissue (Fig. 3b, d, f, h, j). This further demonstrated the stimulatory effect of HMGB1 on the activation of NLRP3 inflammasome. Together these results suggested a novel role for HMGB1 in retinal IR injury by regulating the activation of NLRP3 inflammasomes.

### HMGB1 promoted the processing of IL-1β in elevated IOP-induced retinal IR injury

**Fig. 4:**
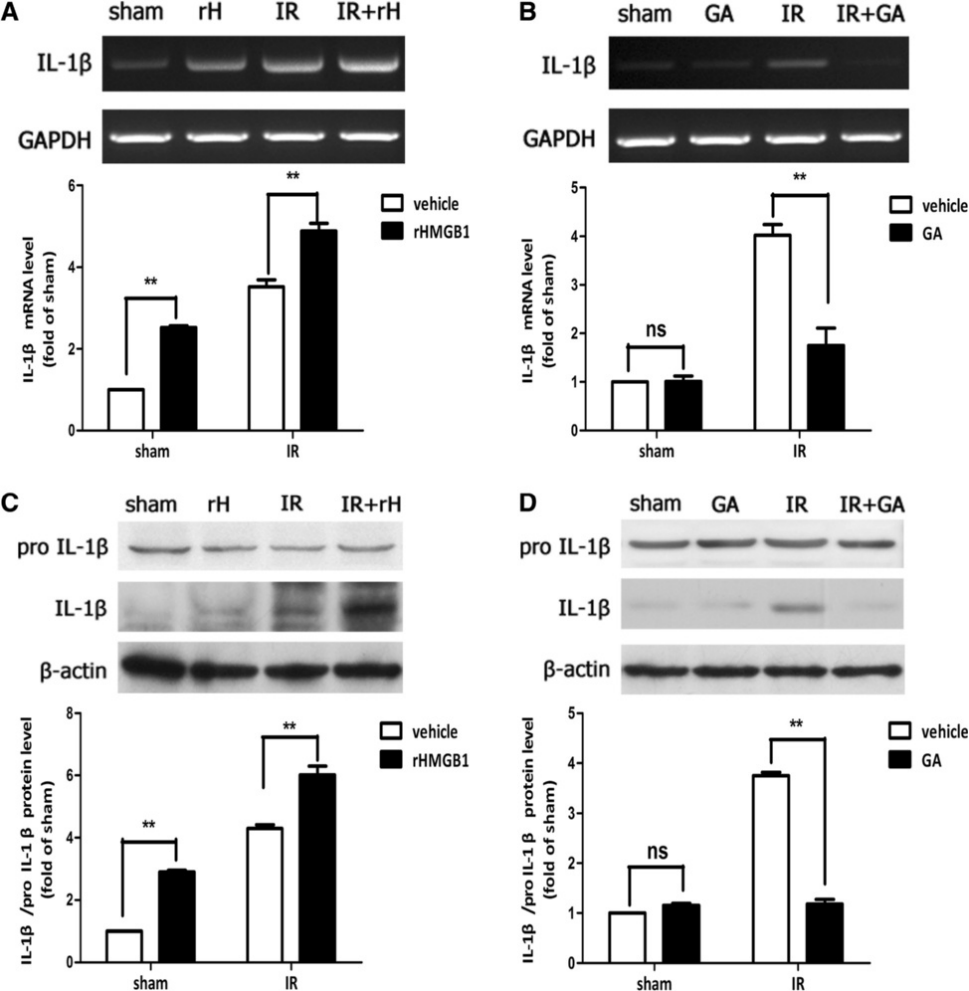
HMGB1 promoted the processing of IL-1β in retinal IR injury. **a**–**d** The intravitreal injection of rHMGB1 or HMGB1 inhibitor significantly enhanced or attenuated the processing of IL-1β in retinal IR injury. Data are shown as mean ± SD. *P < 0.05, **P < 0.01 vs sham group

The stimulatory nature of HMGB1 on the activation of NLRP3 and caspase-8 inflammasomes prompted us to investigate the effect of HMGB1 on the downstream cytokines induced by inflammasomes. Our data demonstrated that the expression of IL-1β was up-regulated in ischemic retinal tissue at both mRNA and protein levels (Fig. 4a, c). The processing of IL-1β was induced or blocked in retinal IR model following rHMGB1 or inhibition by HMGB1 antibody intravitreal injection, respectively (Fig. 4b, d). Overall, these results indicated that HMGB1 release promoted the processing of IL-1β by activating NLRP3 and caspase-8 inflammasomes in retinal ischemic damage.

### HMGB1 regulated NLRP3 and caspase-8 inflammasomes activation and IL-1β production via the NF-κB pathway

**Fig. 5:**
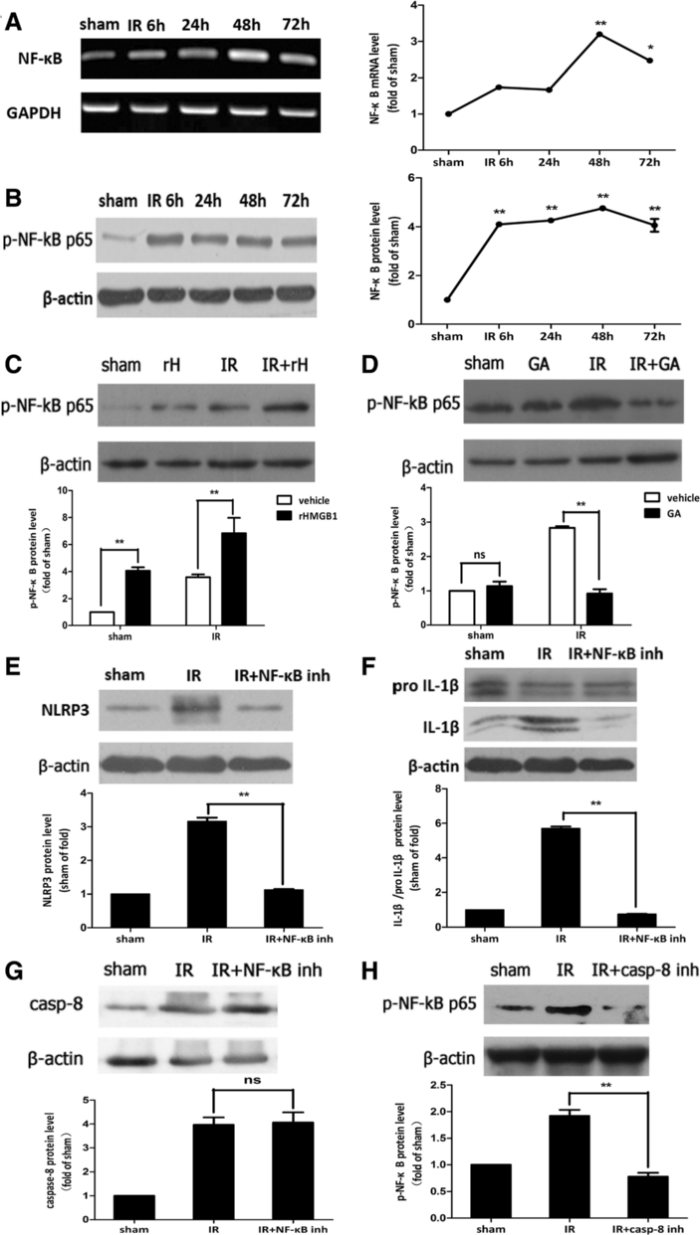
HMGB1 regulated the activation of NLRP3, rather than the activation of caspase-8, via NF-κB pathway. **a**, **b** NF-κB was activated in ischemic retina at the early stage after reperfusion. **c**, **d** Intraocular injection rHMGB1 or GA could promote or suppress the production of phosphor-NF-κB p65. **e**, **f** Intravitreal injection of NF-κB p65 inhibitor, JSH-23, significantly reduced the activation of NLRP3 and decreased the processing of IL-1β, **g** rather than caspase-8. However, intravitreal injection of caspase-8 inhibitor, Z-IETD-fmk, decreased the production of phosphor-NF-κB p65, obviously (**h**). Data are shown as mean ± SD. *P < 0.05, **P < 0.01 vs sham group

Next, we aimed to determine the molecular mechanism by which HMGB1 regulated inflammasome activation and IL-1β production during retinal IR injury. NF-κB p65 mRNA and phosphor-NF-κB p65 protein levels were increased in ischemic retinal tissue (Fig. 5a, b). The level of phosphor-NF-κB p65 increased or decreased to a lesser degree in ischemic retinal tissue in response to the addition of rHMGB1 or HMGB1 inhibitor (Fig. 5c, d), indicating that HMGB1 activated the nuclear translocation of NF-κB in retinal IR injury. To determine if HMGB1 regulated inflammasome activation and induced the processing of IL-1β via NF-κB pathway, the NF-κB p65 inhibitor, JSH-23, was injected into vitreous. Our results demonstrated that the inhibitor of NF-κB significantly reduced the activation of NLRP3 (Fig. 5e) and IL-1β production (Fig. 5f), rather than caspase-8 (Fig. 5g) in retinal IR injury. However, inhibition of caspase-8 could significantly suppress the activation of phosphor-NF-κB (Fig. 5h). All these results demonstrated that caspase-8 was the upstream of NF-κB; HMGB1/caspase-8 pathway induced the activation of NLRP3 and IL-1β production via the NF-κB pathway in retinal IR injury.

## Discussion

Acute glaucoma is a significantly sight-threatening cause of irreversible blindness in the world characterized by a sudden and substantial IOP increase and subsequent RGC death [32, 33]. Acute elevated IOP induced retinal ischemic inflammatory injury, resulting in RGC death. We previously demonstrated that TLR4 promoted the activation of NLRP3 inflammasome which involves in the inflammatory injury of retinal ischemia [27, 34]. However, the role of endogenous ligand of TLR4, HMGB1, is not completely understood. Several studies have reported that NLRP3 promotes the release of HMGB1 and IL-1β [35, 36]. The object of this study is to further explore the influence of HMGB1 on inflammasome activation.

Several reports have demonstrated that HMGB1 plays a critical role in ischemic diseases [8–11]. Likewise, we demonstrated that retinal IR injury triggered the HMGB1 release in retina, accompanying with retinal damage and RGC death. Increase of HMGB1 in retinal IR injury is consistent with the study reported by Dvoriantchikova and Yang [21, 37]. It is well-known that HMGB1 is an endogenous ligand of TLR4. The HMGB1-TLR4 pathway mediates certain ischemic diseases [38, 39]. We also previously demonstrated that TLR4 promotes the retinal ischemic damage via activating caspase-8 signaling and NLRP1 and NLRP3 inflammasomes and processing IL-1β maturation [27]. This prompted us to further explore the mechanism of HMGB1 in mediating retinal IR injury. We injected exogenous HMGB1 or HMGB1 inhibitor into vitreous of retinal IR mice to investigate the biological function of HMGB1 in retinal IR injury. We found that HMGB1 significantly promoted cleaved caspase-8 up-regulation and canonical NLRP3-inflammasome activation, induced the processing of IL-1β, and resulted retinal injury and RGC death eventually.

Traditionally, caspase-8 has been primarily viewed as an initiator of apoptotic cell death activated by receptors of the TNF/NGF family [40–42]. Recently, multiple non-apoptotic roles of caspase-8 have been reported. Importantly, caspase-8 has been reported to have a neuro-inflammatory role [42]. In our previous study, we demonstrated that activation of caspase-8 was involved in the inflammation of ischemic retinal damage via promoting microglia activation, NLRP1/NLRP3 inflammasome activation, processing of IL-1β, and RGC death [27]. In the present study, we firstly provided additional evidence that caspase-8 assembled a novel caspase-8-ASC inflammasome in retinal IR model. Our observation showed that non-cannonical caspase-8 inflammasome was significantly activated in ischemic retinal tissue, which could also been newly found in fungal infection [30, 31]. And the formation of novel caspase-8-ASC inflammasome was relative to the increased HMGB1. Therefore, our data showed that HMGB1 induced the processing of IL-1β via both NLRP3-inducing caspase-1 pathway and non-caspase-1 dependent caspase-8 pathway. Overall, these results indicated that HMGB1 contributed to the inflammation of retinal ischemic damage by promoting the activation of NLRP3 inflammasome, the novel caspase-8 inflammasome, and the processing of IL-1β mediating retinal ischemic damage.

The downstream signaling of all TLR receptors involves three major signaling pathways: mitogen-activated protein kinases (MAPKs), interferon regulatory factors (IRFs), and NF-κB [43]. It has been demonstrated that NF-κB signaling pathway activation was involved in the production of IL-1β in tissue ischemic damage [44]. In the present study, we sought to determine the molecular mechanism of HMGB1 on the processing of IL-1β. Our findings showed that phosphor-NF-κB p65 expression was up-regulated in IR model, and NF-κB signaling pathway activation was associated with increased HMGB1. We further demonstrated that HMGB1 regulated the activation of NLRP3 and induced the processing of IL-1β in a NF-κB-dependent manner. However, caspase-8 was in the upstream of NF-κB, regulating its activation and subsequently the production of IL-1β. Overall, these results demonstrated that HMGB1 regulated the activation of NLRP3, subsequently the processing of IL-1β via NF-κB-pathway and HMGB1 also promoted the activation of caspase-8 which subsequently regulated the activation of NF-κB and then the processing of IL-1β.

In conclusion, our present study showed that HMGB1 release was increased in ischemic retinal tissue as early as 6 h after IR. Exogenous HMGB1 significantly promoted retinal IR injury. We also demonstrated that the novel caspase-8 inflammasome was activated after IR. HMGB1 led to the activation of canonical NLRP3 and non-canonical caspase-8 inflammasomes and the processing of IL-1β in retinal IR injury. In addition, HMGB1 regulated the NLRP3 activation and IL-1β maturation in NF-κB-dependent manner. HMGB1/capase-8 pathway promoted the activation of NF-κB, which subsequently induced the processing of IL-1β. Overall, HMGB1 plays a pivotal role in retinal IR injury through regulating the NLRP3 and caspase-8 inflammasomes activation and IL-1β maturation.

## Abbreviations

AIM2: absent in melanoma 2
ASC: apoptosis-associated speck-like protein containing a CARD
DAMP: a damage-associated molecular pattern
GA: glycyrrhizic acid
GCL: ganglion cell layer
IL-1β: interleukin-1β
INL: inner nuclear layer
IOP: intraocular pressure
IPL: inner plexiform layer
IR: ischemic reperfusion
IRFs: interferon regulatory factors
MAPKs: mitogen-activated protein kinases
NF-κB: nuclear factor κB
NLR: NOD-like receptor
NLRP3: nucleotide-binding domain, leucine-rich repeat containing protein 3
ONL: outer nuclear layer
OPL: outer plexiform layer
RAGEs: receptors for advanced glycation end products
RGCs: retinal ganglion cells
rHMGB1: recombinant high-mobility group box 1
RT-PCR: reverse transcription polymerase chain reaction
TLR: Toll-like receptors
